# Case Report: Immune checkpoint inhibitor-related vitiligo-like depigmentation in non-melanoma advanced cancer: A report of three cases and a pooled analysis of individual patient data

**DOI:** 10.3389/fonc.2022.1099108

**Published:** 2023-01-13

**Authors:** Hui Rao, Zheng Guo, Xuejiao Wen, Xiaoli Zeng, Longqiu Wu, Li Huang

**Affiliations:** ^1^ The First Clinical Medical College, Gannan Medical University, Ganzhou, Jiangxi, China; ^2^ Department of Hematology and Oncology, International Cancer Center, Shenzhen University General Hospital, Shenzhen University Clinical Medical Academy, Shenzhen, Guangdong, China; ^3^ Jiangxi Clinical Medical Research Center for Cancer, Department of Oncology, The First Affiliated Hospital of Gannan Medical University, Ganzhou, China

**Keywords:** immune checkpoint inhibitor, skin adverse events, immunotherapy, vitiligo-like depigmentation, case report

## Abstract

**Background:**

Vitiligo-like depigmentation is a common skin adverse event in patients receiving immunotherapy for malignant melanoma, but has been rarely reported in patients with non-melanoma malignancies. To better understand this immune-related adverse event, we reviewed a series of cases of immunotherapy induced vitiligo-like depigmentation in patients with cancers other than malignant melanoma.

**Case presentation:**

We report three cases of vitiligo-like depigmentation after immune checkpoint inhibitor treatment in gastric adenocarcinoma, lung adenocarcinoma, and squamous cell carcinoma. The first case was treated with camrelizumab, the second was treated with QL1706 injection and sintilimab, and the third was treated with tislelizumab. Pembrolizumab, nivolumab, and ipilimumab caused the majority of vitiligo-like depigmentation, and all three of our patients experienced similar vitiligo-like depigmentation after taking other immune checkpoint inhibitors.

**Methods:**

Three patients who presented with vitiligo-like depigmentation after treatment with immune checkpoint inhibitors were selected. The clinical features, including radiological and histological examination, and the treatment process were reviewed. Eighteen previously published cases of vitiligo-like depigmentation were also used to analyze the results. The severity of vitiligo-like depigmentation in these cases was graded according to the Common Terminology Criteria for Adverse Events, version 5.0.

**Results:**

Vitiligo-like depigmentation occurred in 13 men (61.90%) and 8 women (38.10%), aged from 46 to 79 years, with an average age of 69.9 years. Of the 21 reviewed cases, vitiligo-like depigmentation was described in lung cancer (13/21, 61.90%), clear cell renal cell carcinoma (2/21, 9.52%), acute myeloid leukemia (1/21, 4.76%), cholangiocarcinoma (1/21, 4.76%), urothelial carcinoma (1/21, 4.76%), oral squamous cell carcinoma (1/21, 4.76%), esophageal squamous cell carcinoma (1/21, 4.76%), and gastric adenocarcinoma (1/21, 4.76%). The severity of vitiligo-like depigmentation after immunotherapy was unrelated to sex, age, cancer type, previous autoimmune diseases, and medication.

**Conclusions:**

Vitiligo-like depigmentation is a non-specific skin adverse event in melanoma immunotherapy, but arises as a direct result of treatment with immune checkpoint inhibitors. Vitiligo-like depigmentation has an irregular location, is not limited to direct sunlight cracks, and has also been reported on hair on the head, eyelashes, and eyebrows. People without any skin or autoimmune diseases can also experience vitiligo-like depigmentation after immunotherapy; the incidence of which is irrespective of sex, age, cancer type, previous autoimmune diseases, and medication.

## Introduction

Checkpoint inhibitors such as cytotoxic T lymphocyte-associated antigen-4 (CTLA-4), programmed death protein 1 (PD-1), and programmed death ligand 1 (PD-L1) have been used in the clinical treatment of various tumors. The clinical efficacy of checkpoint inhibitors is accompanied by an increase in immune-related adverse events (irAEs). T-cell activation and subsequent proliferation of inflammatory pathways can lead to a number of immune-related adverse events, of which skin adverse events are the most common. Adverse skin events, including rashes, itching, and vitiligo-like depigmentation, among others (1). Skin toxicity usually occurs in the early stages of treatment, but can also occur days, weeks, or months after treatment (2). Studies have shown that the overall incidence of vitiligo-like depigmentation is approximately 8% when a PD-1 inhibitor is used along with ipilimumab (3); this mainly occurs in patients with malignant melanoma, and is rare in other tumors. Vitiligo-like depigmentation is most common in patients with malignant melanoma, which may be due to the common antigen/T cell cloning of normal melanocytes and tumors (4). Some studies believe that skin adverse events indicate the effectiveness of immunotherapy. Indeed, the appearance of vitiligo-like depigmentation usually indicates that patients with malignant melanoma will benefit from immune checkpoint inhibitors (5, 6). With the exception of malignant melanoma, the relationship between skin toxicity and immunotherapy efficacy in solid tumors remains unknown. Here, we describe three patients with different cancer types (gastric adenocarcinoma, lung adenocarcinoma, and squamous cell carcinoma) who underwent immunotherapy and experienced vitiligo-like depigmentation.

## Case description

### Case 1

**Figure 1:**
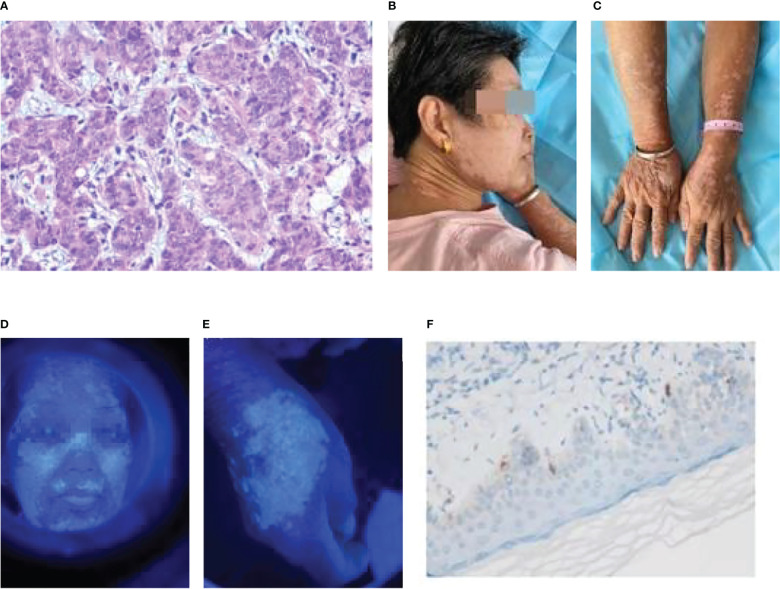
Patient 1: **(A)** Gastric cancer pathology (adenocarcinoma HE×10). **(B)** Vitiligo-like depigmentation of the skin on the face and neck. **(C)** Vitiligo-like depigmentation of the skin on both upper limbs. **(D)** Vitiligo-like depigmentation of the face under the Wood’s lamp. **(E)** Vitiligo-like depigmentation of the right hand under the Wood’s lamp. **(F)** Biopsy pathological immunohistochemistry of vitiligo-like depigmentation (melan-A+).

The first patient, a 64-year-old woman, had gastric adenocarcinoma with systemic bone metastasis (cT4N3M1, stage IV). Cardio adenocarcinoma was identified by pathology (**Figure 1A**), and the immunohistochemistry results were as follows: HER-2 (0); Ki-67, approximately 50% (+); CgA (−); CD56 (−); and Syn (−). The patient was enrolled in a clinical trial, and from April to August 2021, she received six cycles of camrelizumab (SHR-1210) along with oxaliplatin and capecitabine. Chest and total abdominal CT were reexamined every two cycles (6 weeks) to assess the partial response (PR). The patient started to experience body itchiness approximately 1 month after the initial camrelizumab medication, and greater efficacy was observed with loratadine citrate therapy. On June 17, 2021, scattered papules, the largest of which was approximately 3 mm × 3 mm, appeared on the patient’s face and limbs, primarily in the neck and forehead, and it was diagnosed as reactive capillary hyperplasia of the skin. Following the resolution of the papules, patchy vitiligo-like depigmentation appeared on the face, spreading to both forearms, the back of the hand, and neck. The scope gradually widened, and some of the depigmented lesions merged (**Figures 1B** **, C**). Wood’s lamp examination confirmed depigmentation of the patches (**Figures 1D** **, E**). On October 7, 2021, a biopsy of vitiligo-like depigmentation lesions on the back of the patient’s right hand showed hyperkeratosis and hyperkeratosis of skin tissue, superficial dermal edema with vasodilation and congestion of small blood vessels, red blood cell overflow, and lymphocyte and monocyte infiltration in the focal areas. Immunohistochemistry revealed a decrease in melanocytes, and S-100 (+), HMB45 (+), melan-A (+), and approximately 20% Ki-67 (+) (**Figure 1F**). The focus of skin reactive capillary hyperplasia vanished after vitiligo-like depigmentation. The patient had no personal or family history of vitiligo or any other autoimmune disorder.

The patient discontinued camrelizumab in September 2021 because of thrombocytopenia. She withdrew from the clinical trial and was recommended to receive high palliative radiotherapy after targeted therapy with apatinib revealed tumor progression. MRI revealed multiple intracranial metastases due to squabbling after she refused radiotherapy. Whole brain radiotherapy (CTV 30Gy/10fx) was initiated on November 16, 2021 to control brain metastases. On November 29, 2021, a reexamination of the right iliac and lower extremities revealed metastatic cancer. The patient was released on December 2, 2021, with impaired consciousness.

### Case 2

**Figure 2:**
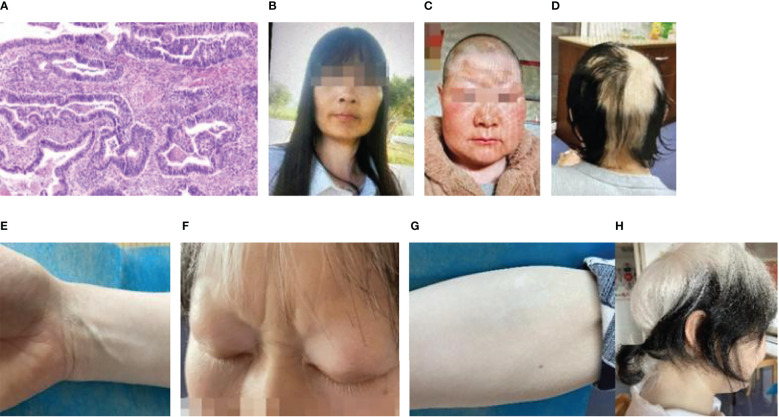
Patient 2: **(A)** Pathology of lung cancer (adenocarcinoma HE×4). **(B)** Photographs of a patient who was diagnosed with vitiligo. **(C)** Photographs of the patient prior to treatment. **(D)** Approximately 2 weeks after the initial treatment, vitiligo-like depigmentation emerged on the skin of the patient’s head and newborn hair. **(E)** Approximately 2 weeks after the initial immunotherapy, vitiligo-like depigmentation began to show on the skin of the patient’s right forearm, comparison to skin tone of the wrist. **(F)** Depigmentation of eyelashes following 12 immunotherapies. **(G)** Significant depigmentation of the skin on the right forearm after the 12 immunotherapies, which gradually fused into fragments and vitiligo-like depigmentation of the skin on the entire arm. **(H)** A side view of the patient following 12 immunotherapies.

The second patient, a 46-year-old woman, had undergone tumor removal of the right front upper mediastinum in the thoracic surgery department of our hospital on April 7, 2016. The right front upper mediastinum’s intermediate differentiated tubular adenocarcinoma was accompanied by necrosis, owing to the postoperative pathology. According to immunohistochemistry, the metastatic middle differentiated adenocarcinoma originated in the intestine. The middle segment of the trachea developed stenosis on August 16, 2016 because of irregular new organisms in the middle and lower segments of the trachea protruding into the lumen. The pathological and immunohistochemical findings supported the diagnosis of adenocarcinoma (**Figure 2A**). Genetic testing revealed wild-type EGFR. Six chemotherapy sessions using pemetrexed and carboplatin were administered from September to December 2016. In August 2018, the patient experienced dull chest and upper abdominal pain, a choking sensation while eating, and face and neck edema. The superior vena cava and mediastinum were both affected by the tumor, which also caused superior vena cava obstruction syndrome and swollen lymph nodes in the mediastinum and right hilum according to a CT scan that revealed the right upper hilum was occupying a space. Pemetrexed and nedaplatin were administered during the two chemotherapy sessions. On November 1, 2018, she received chest intensity-modulated radiation (60Gy/30fx) for visible lung lesions. Pemetrexed and cisplatin treatment was administered concurrently throughout the course of two sessions. On January 11, 2019, CT was reexamined to assess the PR. Pemetrexed was administered for nine courses as part of a maintenance regimen from January to November 2019. CT displayed the progress on January 8, 2020. Pemetrexed plus anlotinib was given on February 29th, paclitaxel plus anlotinib was given on March 28th, docetaxel plus anlotinib was given on March 28th, and paclitaxel plus anlotinib was given for nine courses from May to January 2021, during which the curative effect was confirmed to be stable.

In January 2021, the patient complained of pain in her left hip. The CT scan was reexamined, which revealed multiple metastatic tumors in both lungs. She was accepted into a clinical trial and subsequently received seven courses of immunotherapy with QL1706 (injected) from March to July 2021. Cough, expectoration, and hemoptysis occurred on August 11, 2021, which was accompanied by chest tightness and tightness after exercise. Bronchoscopy revealed irregular new organisms in the lower segment of the trachea, as well as a highly narrow lumen. Most new organisms were clipped out and tracheal stents were implanted using a high-frequency electric snare and hot biopsy forceps. Pemetrexed combined with sintilimab was given for five courses of chemotherapy from September 2021 to January 2022, and pemetrexed was given for four courses of chemotherapy from January to May 2022. The patient’s vitiligo was diagnosed in February 2014, and the lesions were on the left wrist (3 cm × 3 cm), forehead (15 cm × 4 cm), and the neck skin (**Figures 2B** **, C**). Approximately 2 weeks after the first immunotherapy, the original vitiligo focus gradually expanded, and new vitiligo-like depigmentation appeared on the skin of the top of the head (**Figure 2D**), as well as on the skin of the limbs (**Figure 2E**), which fused into pieces. The depigmentation range of the other body parts was significantly expanded, including the hair (on the head, eyelashes, and eyebrows), which also turned white. Thus far, 12 immunotherapies have been administered to the patient, and the depigmentation range has been expanded to include the hair and entire skin of the body (**Figures 2F** **–H**).

### Case 3

**Figure 3:**
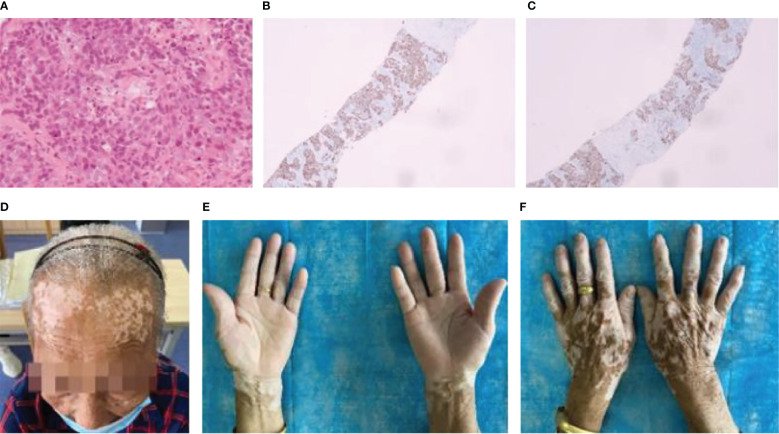
Patient 3: **(A)** Pathology of lung cancer (squamous cell carcinoma HE×10). **(B)** Immunohistochemistry of lung cancer: P40 (+). **(C)** Immunohistochemistry of lung cancer: P63 (+). **(D)** After 12 immunotherapies, the patient’s forehead had vitiligo-like depigmentation and was fused into pieces. **(E)** and **(F)** After 12 immunotherapies, the patient’s hands had vitiligo-like depigmentation.

The third patient was a 79-year-old woman who was diagnosed with squamous cell carcinoma (**Figure 3A**). The results of immunohistochemistry were as follows: CK (+); P40 (+); P63 (+); napsin-A (−); CK7 (−); TTF-1 (−); CgA (−); CD56 (−); Ki-67 (approximately 40%+); Syn (−) (**Figures 3B** **, C**). Six sessions of taxol and carboplatin were administered from November to February 2021. On April 16, 2021, CT examination revealed an increase in the number of intrahepatic metastases, and the curative effect was examined for progressing disease (PD). To address chemotherapy resistance, four sessions of tislelizumab with anlotinib were conducted from April to June 2021. The patient’s forehead and the backs of both hands were covered in vitiligo-like depigmentation, with the largest area measuring approximately 2 cm × 2 cm, around 3 weeks following the start of the first treatment. Immunotherapy caused the dispersed depigmentation sites to fuse into a group and the range gradually widened (**Figures 3D** **–F**), causing depigmentation of a significant proportion of the skin on the waist and belly, resembling vitiligo. The patient had no personal or family history of vitiligo, thyroiditis, underlying skin or autoimmune disorders, recent radiation exposure, and no new medications. On July 15, 2021, CT scan revealed slight growth in some liver metastases since the initial examination, and the curative impact was assessed as stable disease (SD). On schedule, eight cycles of docetaxel chemotherapy, tislelizumab, and anlotinib were administered. The curative effect was assessed as PD on February 26, 2022, when CT scan revealed that the metastatic tumors in the liver were larger than they had previously appeared. On February 28th, March 25th, and April 26th, gemcitabine chemotherapy was administered in three courses, while anlotinib was administered in two courses on March 2nd and April 26th. After discontinuing treatment, the vitiligo-like skin depigmentation remained steady.

## Methods

### Clinical data

This study retrospectively analyzed the clinical features of vitiligo-like depigmentation during immunotherapy, a characteristic cutaneous adverse event in patients with malignant melanoma treated with immunotherapy, which is rare in other malignancies. Currently, approximately 18 cases of vitiligo-like depigmentation have been reported in patients with cancers other than malignant melanoma following immunotherapy. Their age, previous history of autoimmune disease, type of cancer, time to diagnosis, duration of immunotherapy and type of drug, vitiligo-like depigmentation site, and tumor progression were collected. We also included the clinical features of three patients with vitiligo-like depigmentation after immunotherapy at our hospital. The study was approved by the Ethics Committee of the First Affiliated Hospital of Gannan Medical University. Clinical data were collected by reviewing clinical case information and telephone follow-up, including clinical symptoms, imaging findings, and follow-up results.

### Literature research

The literature on immunotherapy complicated by vitiligo-like depigmentation was searched on PubMed and the CNKI database, from inception until March 2022, using the keywords “immunotherapy,” “vitiligo,” and “immune checkpoint inhibitor.” The following selection criteria were used: (I) patients with non-melanoma advanced cancer proven by surgery and pathology; and (II) vitiligo-like depigmentation occurring during immunotherapy. The exclusion criteria were as follows: (I) patients with malignant melanoma or other non-tumor; (II) no immunotherapy given; and (III) no occurrence of vitiligo-like depigmentation during immunotherapy. The authors carefully reviewed the information of each patient, excluding duplicate information. Information on the patient’s age, sex, history of previous autoimmune disease, tumor type, immunotherapy medication, and location and severity of vitiligo-like depigmentation was collected. Information on 18 patients with vitiligo-like depigmentation reported in the literature is shown in **Table 1**.

**Table 1:** Cases of immunotherapy-induced vitiligo-like depigmentation in patients with cancers other than malignant melanoma.

Patient	Age (years)	Sex	Previous autoimmune diseases	Cancer type	Immunotherapy	Position	Other prior medications	No. of treatment lines	PFS (months)	OS (months)	Vitiligo-like depigmentation area severity rating
1 (2)	67	M	No	Lung squamous cell carcinoma	Nivolumab	Frontal, nasal confetti, and the entire skin but preferentially the chest	No	2nd	NA	NA	Grade 2
2 (4)	60	M	Graves’ disease	Clear cell renal cell carcinoma	Nivolumab	Eyebrows, hair, and chest	Sunitinib	2nd	>37	NA	Grade 1
3 (7)	NA	M	No	Oral squamous cell carcinoma	Sintilimab	Hands, feet, head and torso	Paclitaxel and Tegafur	1st	>3	NA	Grade 2
4 (8)	75	F	Hypothyroidism	Non-small-cell lung cancer	Nivolumab	Dorsal hand, ventral forearm, and chin	Carboplatin and pemetrexed	2nd	>22	NA	Grade 1
5 (9)	74	F	No	Esophageal squamous cell carcinoma	Pembrolizumab	Head and neck	No	3rd	33	NA	Grade 1
6 (10)	62	F	Vitiligo	Lung adenocarcinoma	SHR-1210	Aggravated rapidly with depigmentation	Carboplatin and pemetrexed	1st	>22	NA	Grade 2
7 (11)	75	M	Chronic thyroiditis	Lung adenocarcinoma	Nivolumab	Back and thorax	Docetaxel	2nd	>5	NA	Grade 1
8 (12)	63	F	No	Lung adenocarcinoma	Pembrolizumab	Thoracic and back	No	1st	>16	NA	Grade 1
9 (13)	63	M	No	Clear cell renal cell carcinoma	Ipilimumab and nivolumab	Bilateral forearms	No	1st	>27	NA	Grade 1
10 (13)	60	M	No	Cholangiocarcinoma	Pembrolizumab	Bilateral dorsal hands, knees, and lower legs	Cisplatin and gemcitabine	2nd	·NA	NA	Grade 2
11 (13)	79	M	No	Lung squamous cell carcinoma	Pembrolizumab	Arms, back, and lower legs	No	1st	10	13	Grade 2
12 (14)	69	M	No	Lung squamous cell carcinoma	Nivolumab	Face, neck, forearms, hands and trunk	Cisplatin and gemcitabine	4th	>24	NA	Grade 1
13 (15)	61	M	No	Urothelial carcinoma of the right ureter	Atezolizumab	Hands, legs, and scalp	Gemcitabine and cisplatin	2nd	21	>26	Grade 1
14 (16)	75	M	No	Lung squamous cell carcinoma	Pembrolizumab	Belly, waist, hip and upper thighs	No	1st	8	>18	Grade 2
15 (17)	66	M	No	Acute myeloid leukemia	Nivolumab	Bilateral forearms, upper arms, and back	No	2nd	>6	NA	Grade 2
16 (18)	63	M	No	Lung adenocarcinoma	Pembrolizumab	Hands, scrotum, and lower lip	Pemetrexed and cisplatin	2nd	14	NA	Grade 1
17 (19)	72	F	No	Lung adenocarcinoma	Navacizumab	Lip and hairline	No	1st	13	NA	Grade 1
18 (20)	65	M	No	Lung adenocarcinoma	Nivolumab	Right forearm, right upper arm and chest	Carbo AUC-6, pemetrexed, and gemcitabine	3rd	NA	NA	Grade 2
19*	64	F	No	Gastric adenocarcinoma	SHR-1210	face, both forearms, the back of hand and neck	Oxaliplatin and capecitabine	1st	6.1	7.8	Grade 1
20*	46	F	Vitiligo	Lung adenocarcinoma	QL1706 injection and sintilimab	Aggravated rapidly with depigmentation	Pemetrexed, carboplatin, nedaplatin, paclitaxel, anlotinib and docetaxel	4th	6.1	>79	Grade 2
21*	79	F	No	Squamous cell carcinoma	Tislelizumab	Forehead, the backs of both hand, waist and belly	Taxol, carboplatin and anlotinib	2nd	10.5	>24	Grade 2

M, Male; F, Female; ICI, Immune checkpoint inhibitor; NA, No data provided;*Patients treated by the authors; PFS, Progression-free survival; OS, Overall survival.

### Follow-up

The clinical data analyzed in this study, including clinical symptoms, imaging findings, surgical results, immunohistochemical results, and follow-up results, were collected by reviewing the clinical case information and by telephone follow-up. Among these parameters, the clinical history of the patients was collected by retrospective chart review. The potential risk factors assessed included age, sex, history of previous autoimmune disease, tumor type, immunotherapy agents, and the location and severity of vitiligo-like depigmentation.

### Statistical analysis

All statistical analyses were performed using the IBM SPSS statistical package (IBM Corp. version 23.0) using the Fisher’s exact probability method. Significance was set at P < 0.05 in all analyses.

## Results

Following the literature search, we collected data from another 18 patients who received immunotherapy, were complicated with vitiligo-like depigmentation, and had complete data. The severity of depigmented lesions in the 21 patients was graded according to the Common Terminology Criteria for Adverse Events, version 5.0 (21). The specific criteria were as follows: grade 1, hypopigmentation or depigmentation covering <10% body surface area (BSA), and no psychosocial impact; grade 2, hypopigmentation or depigmentation covering >10% of the BSA and an associated psychosocial impact. Results of previous reported cases in the literature are shown in **Table 1**. In the case of our hospital, patients with gastric adenocarcinoma were classified into grade 1, those with lung adenocarcinoma or lung squamous cell carcinoma were classified into grade 2. The results of the statistical analysis are shown in **Table 2**. Vitiligo-like depigmentation occurred in 13 men (61.90%) and 8 women (38.10%), aged from 46 to 79 years, with an average age of 69.9 years. Vitiligo-like depigmentation has been described in lung cancer (13/21, 61.90%), clear cell renal cell carcinoma (2/21, 9.52%), acute myeloid leukemia (1/21, 4.76%), cholangiocarcinoma (1/21, 4.76%), urothelial carcinoma (1/21, 4.76%), oral squamous cell carcinoma (1/21, 4.76%), esophageal squamous cell carcinoma (1/21, 4.76%), and gastric adenocarcinoma (1/21, 4.76%) (2, 4, 7–20). The severity of vitiligo-like depigmentation after the use of immunotherapy was not related to sex, age, cancer type, previous autoimmune diseases, or medication(P>0.05, **Table 2**).

**Table 2:** Relationship between the severity of vitiligo-like depigmentation and clinical characteristics in the pooled 21 cases.

Value	Classification	G1	G2	P-value
Sex	Male	6	7	0.659
	Female	5	3	
Cancer type	Lung cancer	6	7	0.659
	Non-lung cancer	5	3	
Age (years)	≥75	2	3	0.918
	<75	9	6	
	NA	0	1	
Previous autoimmune diseases	Vitiligo	0	2	0.214
	Other 1	11	8	
Immunotherapy	Nivolumab	5	3	0.809
	Pembrolizumab	3	3	
	Other 2	3	4	
Other prior medications	Yes	7	6	1
	No	4	4	

NA, No data provided; Other1, Other autoimmune diseases besides vitiligo or no autoimmune diseases; Other2, Other immune checkpoint inhibitors besides nivolumab and pembrolizumab.

## Discussion

Vitiligo is an autoimmune disease characterized by depigmented patches or macules on the skin. Typically, lesions in nonsegmental vitiligo are symmetric, confluent, snow-white patches, with well-demarcated borders. They can be widely dispersed, preferring the face, dorsa of the hands and feet, trunk, anogenital regions, elbows, knees, axillae, inguinal folds, and forearms, among other sites (22). However, vitiligo-like depigmentation is an adverse skin event of immunotherapy that is less progressive, asymmetric, and limited in size, with a variable and more atypical distribution than classic vitiligo, which is confined to a light-exposed area, with several spots and asymmetry (1). This view is supported by the patient with gastric cancer in this study, whose depigmentation was primarily found on her face, forearm, backs of hands, and neck. However, the depigmentation sites of lung adenocarcinoma and squamous cell carcinomas are irregular. In the patient with lung adenocarcinoma, vitiligo-like depigmentation also occurred in places that are not exposed to light, such as the upper arm, chest skin, and perineum hair. The patient’s hair, eyebrows, and eyelashes are all exposed to light. The depigmentation in the patients with squamous cell carcinoma occurs in places that are not exposed to light, such as the waist and abdomen, indicating that the vitiligo-like depigmentation caused by immune checkpoint inhibitors is not only a multi-spotted lesion but also a fusion phenomenon. Simultaneously, other articles have noted that the depigmentation sites of patients with malignant tumor with nonmalignant melanoma are primarily distributed in the back, chest, and lower limbs, and are not restricted to places that receive direct sunshine (11, 13). The depigmentation in varied body areas, including the hair, suggests that the location and severity of this adverse event are not regular or predictable. Each patient had at least two areas of depigmentation. In most reports, depigmentation was located remotely from the primary tumor location, without the involvement of the adjacent skin (**Table 1**). A previous case report described a patient with intestinal cancer who developed pseudo-vitiligo following two cycles of capecitabine treatment despite having no history of skin problems. The skin lesions were characterized by hypopigmentation on both hands. The hypopigmentation slowly recovered after stopping capecitabine (23). Nevertheless, the vitiligo-like depigmentation brought on by immune checkpoint inhibitor medication did not go away after discontinuing immunotherapy, and may even have worsened, suggesting a continued effect of immunotherapy. After stopping camrelizumab medication for more than 40 days, the patient with gastric cancer showed new skin depigmentation in the chest region. Additionally, the skin lesions persisted even after immunotherapy was stopped, suggesting that immunotherapy may cause irreversible skin manifestation. However, the current cases have limitations in determining the cancer prognosis and reversibility of vitiligo-like depigmentation.

Vitiligo is caused by the loss of melanocytes and is linked to many hereditary and immunological illnesses (5). Our literature review revealed that many patients suffered from vitiligo-like depigmentation after receiving immunotherapy. Moreover, many of these patients had no previous personal or family history of vitiligo or other autoimmune diseases, indicating that vitiligo-like depigmentation occurs independently of previous skin or autoimmune diseases, and is not associated with age or sex. Among the three cases in this study, those with lung squamous cell carcinoma and gastric cancer had no history of autoimmune disorders or skin conditions. In the patient with lung adenocarcinoma, the original skin depigmentation site of the lung adenocarcinoma spread to the entire body after immunotherapy, with depigmentation even observed in the hair and other areas. Melanocyte destruction leads to vitiligo-like depigmentation in patients with malignant melanoma after immunotherapy, possibly because normal melanocytes share antigen-responsive T cell cloning with tumors (24). The occurrence of vitiligo-like depigmentation in other patients with tumors indicates a distinct pathogenesis from malignant melanoma. A study has reported a case of autoimmune pemphigus vulgaris in a patient treated with cemiplimab for multiple locally advanced cutaneous squamous cell carcinoma with good control of skin disease with steroid therapy and no negative effect on outcomes. This study indicated that monoclonal antibodies that target PD-1/PDL-1 pathways may induce immune-mediated adverse events possibly related to a reduction in regulatory T cells, leading to increased T-cell activation, B-cell proliferation, and synthesis of autoantibodies. These immunological mechanism both enhance the antitumoral response and unleash the onset of autoimmune diseases in predisposed subjects (25). Some studies have suggested that immunotherapy significantly increases the infiltration of CD8+T cells expressing CXCR3 and the level of serum CXCL10 in vitiligo-like depigmentation; this phenomenon was not observed in healthy subjects, indicating that this cell subset may play a role in depigmentation (22).

Vitiligo-like depigmentation in patients with malignant melanoma undergoing immunotherapy is usually positively connected with curative outcome (26–28), but its relationship with curative impact on other solid tumors is yet unknown. The progression-free survival (PFS) and overall survival (OS) in patients with gastric adenocarcinoma was only 6.1 and 7.8 months, respectively, compared to the results of first-line chemotherapy combined with immunotherapy in patients with advanced gastric cancer in CheckMate-649 (29) and the Asian study ATTRACTION-4 (30), in which the PFS and OS did not improve significantly. The PFS for patients with lung adenocarcinoma with QL1706 was 6.1 months, that for patients treated with sintilimab was 9.9 months, and their OS was more than 79 months. The PFS in the patient with squamous cell carcinoma was 10.5 months and their OS was more than 24 months. Based on CheckMate-078 (31), RATIONALE-303 (32), and OAK studies (33), these two patients had a better significant prognosis, and follow-up is still ongoing. We will continue to track the changes in depigmentation in these patients in the future, and, if appropriate, obtain histological information of the extended depigmentation to confirm the pathogenesis. By gathering more data, it will be possible to forecast the outcomes of patients and better understand the connection between vitiligo-like depigmentation and immunotherapy.

Clinicians must assess patients’ susceptibility to skin toxicity at the start of immune checkpoint inhibitor therapy, educate patients, and thoroughly enquire about the patient’s history of autoimmune, endocrine, and infectious diseases, family history, previous anti-tumor drug treatment, and baseline medications. Simultaneously, the skin and mucosa of patients should be examined, particularly for patients with a history of autoimmune disease of the skin. Furthermore, as the onset time of skin toxicity remains unknown, it is crucial that patients promptly notify the treatment team of any suspicious symptoms and seek medical attention. Based on symptoms and signs, laboratory tests and examinations can accurately assess the severity. Most cases of skin toxicity can be prevented by appropriate intervention measures without affecting the continued use of immune checkpoint inhibitors providing that the toxicity is detected early and the patient receives timely intervention by clinicians. Vitiligo-like depigmentation is often regarded as a G1, G2 immune-related toxic reaction, and oral glucocorticoids can generally be used for treatment (34). The effectiveness of immunotherapy may be negatively impacted by the use of high dose glucocorticoids due to skin toxicity; however, there is currently no conclusive clinical evidence to support this claim. The use of corticosteroids to treat symptoms brought on by malignant tumors had a negative impact on patients’ PFS and OS, but the use of steroids to treat irAEs did not appear to have a negative effect on patients’ prognosis, according to a retrospective cohort study of patients with metastatic non-small-cell lung cancer who were treated with immune checkpoint inhibitors (35). All three of our cases elected for close monitoring with routine skin surveillance and no additional treatment, and the immunotherapy plan has not been altered or discontinued.

Vitiligo is not life-threatening, but exerts a harmful influence on the quality of life. Patients who have depigmentation may have inferiority complexes, shame, melancholy, and social isolation, all of which could result in mental illnesses and have a negative impact on patients’ health and quality of life, including anxiety, depression, and even suicidal behavior (3). Establishing a harmonious relationship with patients and raising the awareness that vitiligo-like depigmentation is not contagious will reduce the humiliation and discrimination of patients because of their appearance and help to improve their quality of life.

## Conclusion

Vitiligo-like depigmentation is a non-specific skin adverse event in immunotherapy for malignant melanoma, but a direct reaction after treatment with immune checkpoint inhibitors. This kind of adverse event occurs irregularly, not only in the area exposed to direct sunlight, but also in areas such as the hair and eyelashes. Patients with no history of skin or autoimmune diseases may suffer from vitiligo-like depigmentation after immunotherapy. Most vitiligo-like depigmentation in patients with malignant melanoma follows treatment with pembrolizumab, nivolumab, and ipilimumab; however, no such pattern has been observed with other tumors, and depigmentation may occur after treatment with any immune checkpoint inhibitor as a direct adverse event of immunotherapy. Vitiligo-like depigmentation does not go away when immunotherapy is stopped, and even has the potential to worsen. At present, the relationship between the occurrence of such adverse events in patients with other tumor types and the efficacy of immunotherapy is unclear, and a collection of more cases will help physicians to better understand the association between vitiligo-like depigmentation and immunotherapy.

## Data availability statement

The datasets presented in this study can be found in online repositories. The names of the repository/repositories and accession number(s) can be found in the article/supplementary material.

## Ethics statement

The studies involving human participants were reviewed and approved by the Ethics Committee of the First Affiliated Hospital of Gannan Medical University. The patients/participants provided their written informed consent to participate in this study. Written informed consent was obtained from the individual(s) for the publication of any potentially identifiable images or data included in this article.

## Author contributions

HR and LH: conception and design of the study. HR: manuscript writing and sample collection. ZG: manuscript review and data analysis. XW, XZ, and LW: manuscript review, resident in charge of patients during treatment. LH: supervision of manuscript and patient treatment. All authors contributed to the article and approved the submitted version.
